# The final frontier: using carcasses for one health surveillance at the ecosystem interface

**DOI:** 10.3389/fvets.2025.1683110

**Published:** 2025-11-28

**Authors:** Katie A. Barton, Patrick B. Finnerty, Ruwini Rupasinghe, Carlos González-Crespo, Jackie E. Mahar, John-Sebastian Eden, Niraj Y. Meisuria, Beatriz Martínez-López, Thomas M. Newsome, Alison J. Peel, Justine A. Smith, Victoria J. Brookes

**Affiliations:** 1 Sydney School of Veterinary Science, Faculty of Science, The University of Sydney, Camperdown, NSW, Australia; 2 School of Life and Environmental Sciences, Faculty of Science, The University of Sydney, Camperdown, NSW, Australia; 3 Center for Animal Disease Modeling and Surveillance, University of California, Davis, Davis, CA, United States; 4 Australian Animal Health Laboratory and Health and Biosecurity, Commonwealth Scientific and Industrial Research Organisation, Geelong, VIC, Australia; 5 Centre for Virus Research, Westmead Institute for Medical Research, Westmead, NSW, Australia; 6 Sydney Infectious Diseases Institute, The University of Sydney, Camperdown, NSW, Australia; 7 Department of Wildlife, Fish, and Conservation Biology, University of California, Davis, Davis, CA, United States

**Keywords:** scavenging, infectious disease, genomic, spillover, carrion, risk

## Abstract

Anthropogenic activities such as agricultural intensification, urbanisation, globalisation, and climate change are accelerating disease emergence globally, yet surveillance systems have largely overlooked the critical role of vertebrate carcasses in pathogen transmission. This omission is concerning because animal mass mortality events (MMEs) are increasing in frequency and magnitude, while populations of key vertebrate scavengers, especially obligate scavengers like vultures, are declining, resulting in longer carcass persistence and altered disease risks. Carcasses serve as essential resources in food webs but also act as complex microbe transmission hubs through direct consumption, environmental contamination, vector-mediated dispersal, and increased host aggregation, facilitating cross-species and trophic spillover events. Scavengers can amplify or mitigate microbe transmission: their consumption of carcasses can remove infectious material, but their mobility and sociality may also disperse potential pathogens across large areas. Technological advances, including remote sensing, camera traps, GPS telemetry, and machine learning, now enable detailed tracking of scavenger-carcass interactions and identification of transmission hotspots. Simultaneously, metagenomic sequencing allows untargeted detection of known and novel pathogens in carcass-associated microbial communities (“necrobiome”), with portable platforms supporting field-based surveillance. Integrating carcass-based surveillance into One Health frameworks through interdisciplinary collaboration among ecologists, epidemiologists, and data scientists offers a proactive approach to early outbreak detection, improved pandemic preparedness, and ecosystem health monitoring. Given the projected increase in climate-driven mortality events, incorporating carcass-scavenger networks into disease surveillance strategies is a valuable and under-utilised complement to existing approaches, enhancing our ability to monitor and mitigate emerging infectious diseases.

## 1 Introduction

Disease emergence has accelerated in recent decades, driven by anthropogenic activities such as intensification of agriculture and increasing urbanisation, which exacerbate climate change effects and degrade natural ecosystems (1). As these pressures reshape the boundaries between ecosystems, wildlife, humans, and domestic animals, understanding how diseases emerge at the intersections of these systems (the animal–human–ecosystem interface) is a central focus in infectious disease research. Nevertheless, research has largely focused on interfaces involving living animals (2, 3). The interface between vertebrate carcasses and ecosystems has been largely overlooked, however, carcasses may provide an opportunity for pathogen enrichment, transmission and disease emergence, either from direct contact with scavengers or through species congregating at carcasses (4).

Adopting a One Health perspective, which recognises the interconnectedness of human, animal, and environmental health, underscores the importance of considering all facets of the animal–ecosystem interface, including the often-overlooked role of carcasses (5, 6). In this context, carcass degradation serves not only as a potential vector for pathogen transmission but also as a valuable indicator of ecosystem health (7). Carcass decay is a key ecological process, driven by intricate interactions among scavengers and microbes that limit biomass accumulation by consuming tissue, breaking down organic matter, and redistributing and recycling nutrients across ecosystems (7). Disruptions to the decomposition process can indicate ecosystem imbalances, including shifts in scavenger communities (scavenger guilds) or changes in carcass availability (8).

We argue that carcasses are an under-utilised resource for One Health surveillance, offering not only insights into disease risk, but also as critical indicators of ecosystem health. Here, we synthesise current knowledge on scavenger interactions with terrestrial vertebrate carcasses (including marine mammals), examine the associated disease risks and transmission pathways, and highlight how carcasses can be harnessed for proactively monitoring emerging and re-emerging infectious diseases within an integrated One Health framework.

## 2 Increasing carcass availability: carcass booms and scavenger bursts

Carcass availability is changing in some areas due to an increase in mass mortality events (MMEs; also known as population die-offs) and changes in scavenger populations (9, 10). An MME is a phenomenon which has been defined as the sudden death of a large percentage of a population of animals in a short period relative to the species’ generation time (10). From an epidemiological perspective, an MME could also be defined in terms of an outbreak, i.e., an increase in the expected number of deaths in a defined time period, location, and population (11). However, this framing often fails to capture the massive scale and impact of MMEs, which can decimate populations. For instance, an MME in critically endangered saiga antelope (*Saiga tatarica*) in Kazakhstan in May 2015 resulted in the death of over 200,000 individuals in just three weeks, eliminating >60% of the global population (12). Similarly, in 2020, over 350 African elephants (*Loxodonta africana*) were found dead in Botswana’s Okavango Delta over a matter of weeks, believed to be linked to cyanobacterial toxins (13, 14). Disease has also been implicated in MMEs that resulted in rapid population collapses of wildebeest (*Connochaetes* spp.) (15) and vicuñas (*Vicugna vicugna*) (16).

Catastrophic MMEs are projected to become more frequent in the coming decades because they are often triggered by disease outbreaks, environmental stressors such as extreme weather events, and the need to actively manage invasive species, each of which are increasing in intensity and frequency across many global regions (17–21). A current example is the global spread of H5N1 high pathogenicity avian influenza (HPAI) virus. Clade 2.3.4.4b H5N1 HPAI virus has caused outbreaks and mass mortality in many waterbird species globally, and since 2022, has killed thousands of marine mammals in South America following development of mammal-to-mammal transmission (22, 23). Extreme weather is increasingly contributing to MME events, particularly when limited connectivity inhibits animals from escaping lethal environmental conditions (24). In some cases, drivers of MMEs are synergistic; in the saiga antelope MME, mortality was caused by haemorrhagic septicaemia, triggered by extreme humidity and elevated temperatures that heightened host susceptibility to *Pasteurella multocida* type B, a normally commensal bacterium (12). Intentional MMEs are also on the rise as mass culling of wildlife is increasingly used to control invasive species (21, 25). For example, in Australia, large populations of introduced feral herbivores such as horses, deer, and camels are culled to reduce environmental degradation, with carcasses left *in situ* (26–28).

A single MME can generate millions of tonnes of carcass biomass, dramatically increasing the amount of carrion in affected ecosystems (17). Such surges in necromass can overwhelm scavenger communities, altering complex scavenging dynamics and influencing disease risks (see below) (9). Increases in carcass biomass can also be exacerbated by shifts in scavenger guilds, including scavenger population declines and contractions in geographic ranges. A key example is the decline in global vulture populations in recent decades, nearing extinction in some Asian and African countries (29). In South Asia, the livestock carcasses that previously sustained vulture populations became lethal: the widespread use of the veterinary drug diclofenac was identified as a primary driver of decline, because vultures, which are obligate scavengers, died of renal failure after consuming treated carcasses (30). This decline created an ecological cascade whereby reduced scavenging efficiency led to livestock carcass accumulation and increased disease transmission risks through expansion of alternative scavengers, such as feral dogs (31). Additional pressures, including changes in legislation, habitat destruction and both intentional and accidental poisoning, have further accelerated vulture declines worldwide (29, 32, 33).

Compounding this, apex carnivores are in decline globally (34) with significant cascading effects across trophic levels (35). Healthy apex carnivore populations maintain scavenger communities by providing carrion year-round rather than in seasonal pulses (36). Although declines of dominant carnivore scavengers can facilitate scavenging by subordinate carnivores, they can rarely replace their functional roles in terms of their ability to rapidly consume carrion (37). Rapid declines in apex carnivore populations are thus likely to further disrupt the structure and function of scavenger communities worldwide (38).

## 3 Scavenger activity and pathogen risk

**Figure 1:**
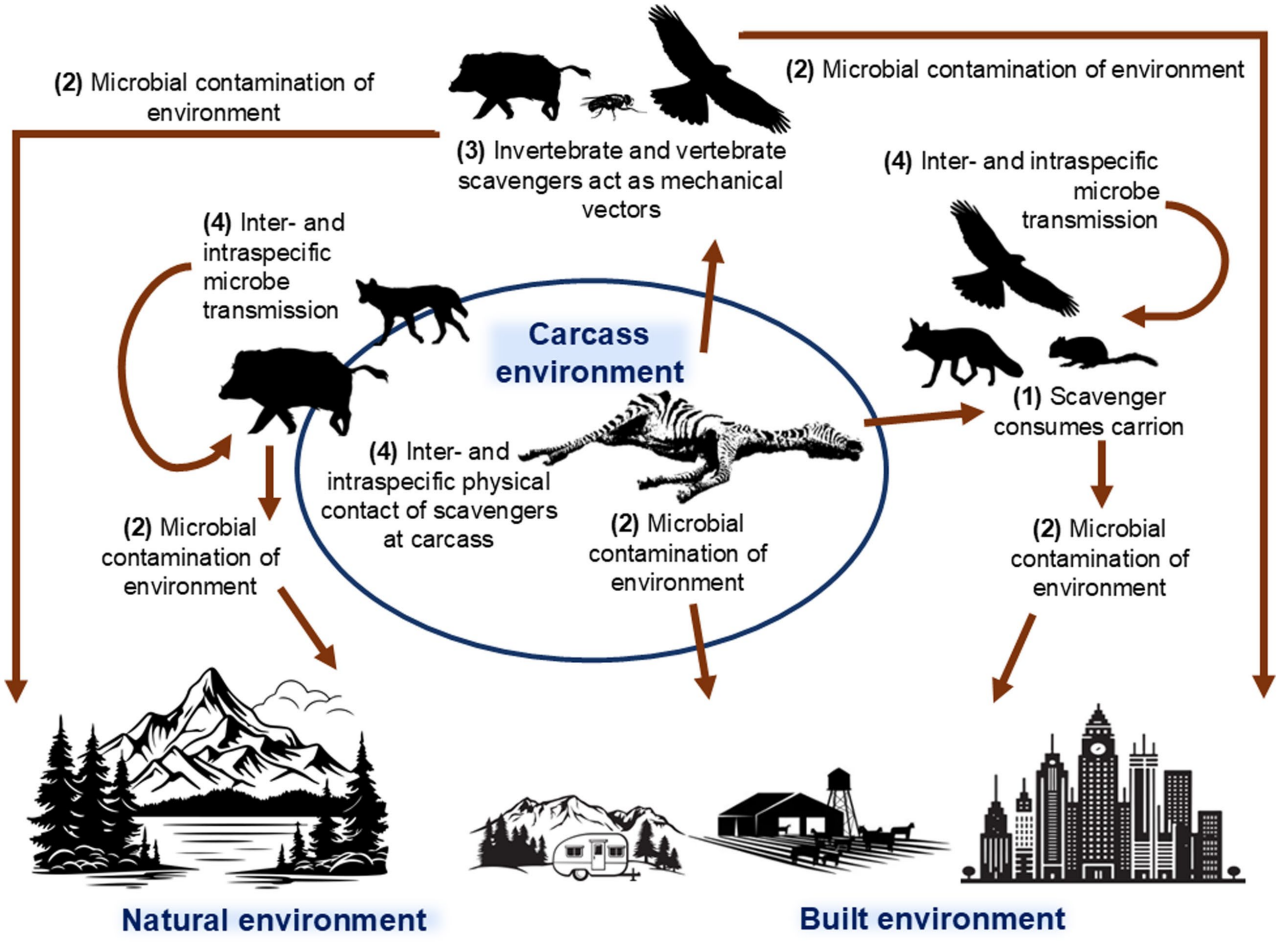
Conceptual diagram of the routes of microbial spread associated with carcasses, scavengers and the environment. Numbers are used to denote the four potential routes for pathogen transmission and arrows indicate the direction of microbial movement. Route 1: Direct ingestion—scavengers acquire microbes by consuming carcasses. Scavengers can become infected with potential ongoing inter- and intraspecific transmission and environmental contamination (Route 2, for example by faecal contamination), or remove microbes thus reducing opportunity for microbial spread. Route 2: Environmental contamination—microbes are released into the environment either directly into the soil, water, or vegetation, or indirectly via scavengers, potentially contaminating the broader ecosystem including built environments. Route 3: Vector-mediated microbial spread from carcasses, potentially over large distances. Route 4: Inter- and intraspecific interactions that occur between scavenger species attracted to carcasses that increase direct contact, competition, and predation.

Carcasses present multiple potential pathogen transmission opportunities via a range of routes for microbial spread (Figure 1), including direct consumption of infected carrion, environmental contamination from the carcasses or visiting scavengers, vector-mediated transmission, transmission associated with increased scavenger host aggregation and contact, and trophic interactions with microbial spillover. Scavengers play a dual role in these processes, amplifying or mitigating microbe spread depending on microbe characteristics, scavenger species, and ecosystem context (39–42).

Firstly, direct ingestion of microbes through scavenging (Route 1, Figure 1) is a critical transmission route for pathogens associated with multiple zoonotic and wildlife diseases. Key examples include: highly pathogenic avian influenza virus (HPAI H5N1) via consumption of infected bird carcasses by raptors and mammals (43); anthrax (sporulation occurs after scavengers open a freshly infected carcass and expose it to air, allowing spores to form and be disseminated) and tuberculosis in scavengers such as hyaenas and vultures (44, 45); botulism following neurotoxin uptake from decomposing carcasses, especially by waterbirds (46); and African swine fever (ASF) from consumption of ASF-infected conspecific carcasses (47). Alternatively, consumption can remove infectious carrion from the environment and reduce ongoing transmission risk. For example, vultures achieve this through rapid consumption of carcasses and their acidic gastric environment, which inactivates pathogens and limits carcass persistence (42, 48).

Secondly, environmental contamination can occur directly from carcasses or indirectly via scavengers (Route 2, Figure 1). As carcasses decompose, they can release microbes into the soil, water, or vegetation, creating long-term environmental reservoirs for ongoing transmission. Examples include chronic wasting disease (CWD) in which prions bind to soil minerals and remain infectious for years, or anthrax spores that persist in soil for over a decade, infecting herbivores via contaminated grazing sites (49, 50). Similarly, a wild boar carcass infected with the ASF virus can contaminate the surrounding soil for approximately one month (51).

Thirdly, vector-mediated routes (Routes 3, Figure 1) can occur via a range of scavengers. Scavengers typically use extensive areas of space (52), enabling microbe spread from a carcass over large geographical regions via infection of the scavenger, movement of carrion, defecation, or contamination of fur, feathers, or feet. A previous study in Germany found that wild boar carcasses, a potential source of ASF virus, were scavenged and dispersed up to 1.2 km away from the initial carcass site (53). Blowflies can carry anthrax spores from infected carcasses and deposit them onto surrounding vegetation, thereby creating new foci of infection for grazing herbivores (54). Invertebrates are also known to transmit *Clostridium botulinum* type C toxin from decomposing carcasses to vertebrate species (55).

Finally, as critical nutrient sources, carcasses can attract diverse scavenger species, promoting and intensifying both inter- and intraspecific interactions, including competition and intraguild predation (Route 4, Figure 1) (56). This can facilitate disease transmission via direct contact; for example, rabies transmission has been documented among jackals congregating at anthrax-generated carcasses (57). Similarly, bovine tuberculosis can be transmitted between mammals at shared feeding sites (44). These congregation events not only elevate the risk of direct pathogen transfer among conspecifics but also increase the likelihood of cross-species transmission. Scavengers that become infected at carcass sites may also subsequently transmit pathogens to new hosts through predation, social contact, or other ecological interactions. For example, wolves preying on or scavenging CWD-infected coyotes could facilitate the movement of prions across trophic levels and geographic boundaries (58). Similarly, mammalian scavengers such as feral dogs or pigs can act as ecological bridges between wild and domestic animals, enabling the spillover of pathogens like ASF from wildlife reservoirs into livestock populations (59).

## 4 Who goes there? Characterising the carcass-scavenger interface

Effective outbreak mitigation strategies require detailed knowledge of the mechanisms by which species interact and transmit microbes at these interfaces. Therefore, knowledge of carcass–scavenger dynamics is essential for effective integration of carcass-based surveillance into One Health frameworks. Multiple factors influence microbial transmission at the carcass-scavenger interface; for example, virus longevity in carcass material is important, and evidence suggests that initial scavengers, particularly in winter, are most at risk of infection and most likely to contribute to disease transmission (44, 60, 61). Historically, field researchers relied on direct observation and systematic carcass surveys to identify scavenger species, estimate carcass persistence, and document scavenging events (62). While valuable, these methods are labour-intensive, can disturb wildlife, and often miss nocturnal or elusive scavengers.

Recent advances in technology and analytical methods have greatly enhanced our ability to monitor, quantify, and model these complex ecological and epidemiological processes. Remote-sensing technologies, particularly camera traps and GPS collars, have transformed scavenger research by enabling continuous, non-invasive monitoring across spatial and temporal scales. Camera traps deployed at carcasses provide comprehensive empirical data on scavenger communities, documenting species identities, individual behaviours, visitation patterns, scavenging rates, and temporal dynamics (63–67). Camera-traps also capture inter- and intraspecific interactions, including aggressive encounters and feeding hierarchies, and have revealed scavenging behaviours in species not previously recognised as key scavengers (68, 69). GPS collars complement these data by providing fine-scale information on scavenger movement across landscapes, including home range size, movement corridors, timing and frequency of carcass visits, intraspecific interactions, and the potential for long-distance pathogen dispersal (52, 70, 71).

In parallel with technological advances, there is an increase in computational power and use of machine learning methods to process large amounts of data. For example, automated processing of camera trap images to identify species and behaviours can enable detection of unusual events such as potential disease outbreaks (72, 73). Whilst the carcass-scavenger interface has predominantly been investigated using statistical models (63, 64, 66), relatively under-utilised analytic methods such as network analyses can be used to investigate complex carcass-scavenger-microbe dynamics with opportunities for dynamic simulation of microbial transmission in populations associated with carcasses (74). Modelling such interactions can identify the key species, individuals, or carcass sites that act as “superspreaders” or transmission hubs, quantify the probability and directionality of disease transmission pathways, and simulate the effects of interventions (for example, targeted carcass removal, vaccination, or culling) on disease dynamics and ecosystem function (8, 65, 75–78). Importantly, such models can inform outbreak responses that mitigate disease risk while preserving scavengers essential ecological functions. Combining scavenger movement and contact data with environmental sampling (for example, on the carcass itself, and soil, water, and vegetation) would enable a holistic view of microbial succession, pathogen persistence, and ecosystem health at carcass sites. Widespread adoption of this comprehensive approach would strengthen the role of carcass-based surveillance within One Health frameworks by providing opportunity for the development of effective disease risk mitigation strategies.

## 5 Communicating with the necrobiome: tools and technologies for pathogen detection

To understand the disease risks associated with carcasses, it is essential to identify and characterise the necrobiome (the microbial community associated with carcasses) and integrate this knowledge into a broader surveillance framework. Traditional pathogen detection methods such as polymerase chain reaction (PCR), serological assays like ELISA, and virus isolation through cell culture have been widely used (79, 80). However, these approaches are limited to detecting known, specifically-targeted pathogens, either directly (via microbial genetic material or particles) or indirectly (via host immune responses such as antibodies) and are often labour-intensive (81). Given the concern of emerging infectious diseases (EIDs), particularly those that are novel or unknown, surveillance strategies must be able to interrogate microbial communities in an unbiased manner. Advances in high-throughput sequencing technologies and bioinformatics, sophisticated computational and statistical models, and increasing computing power allow for ‘omics-based analyses, which offer comprehensive profiling of microbial diversity and strong potential for novel pathogen discovery, without the need for microbial isolation and laboratory cultivation (71, 80, 82–84).

Among these analyses, metagenomic sequencing allows for the untargeted analysis of all genetic material present in a sample, enabling identification of both known and previously undescribed pathogens (85). This approach has demonstrated its utility in surveillance settings, including in the investigation of carcasses following a mass mortality event (86, 87). Although the application of metagenomics in that event was reactive (i.e., associated with a response investigation, not surveillance), it underscores the feasibility and relevance of metagenomics in carcass-based surveillance (86). Similarly, metatranscriptomics (a specific type of metagenomics in which only RNA is sequenced) enables untargeted investigation of the RNA profile of a sample, providing insight into microbial gene expression, abundance, and the active replication state of the host or microbial community (88). Importantly, this approach is highly effective for RNA virus detection, and facilitates detection of actively replicating DNA viruses, bacteria, fungi, and parasites and has proven utility for pathogen discovery, surveillance, and infectome characterisation (compared to metagenomic approaches in which only DNA is sequenced) (89–92). This is particularly important in the context of EIDs, because RNA viruses—known for their high mutation rates and pandemic potential—are a frequent source of zoonotic spillover (93). Additionally, metatranscriptomics and metagenomics allow species- and strain-level classification of pathogens and can provide insights into the functional potential of a microbial community (94). Marker gene amplicon sequencing (for example, targeting 16S rRNA in bacteria, ITS/18S for fungi/parasites) offers a cost-effective and rapid alternative to more expensive, time-consuming methods for taxonomic profiling of non-viral microbial communities. However, this approach is inherently targeted, provides limited functional insights, and lacks universal targets for viruses. While family-specific PCR assays exist for some viruses (for example, paramyxoviruses, coronaviruses), these only amplify small genomic regions, resulting in insufficient resolution for detailed strain typing (95, 96). Consequently, RNA-based metagenomics remains the essential method for comprehensively capturing the entire microbial diversity within a system.

## 6 Conclusion

Carcasses and their associated scavenger networks present a growing challenge to biosecurity, zoonotic spillover prevention, and global pandemic preparedness. As focal points where wildlife, microbes, and the environment intersect, carcass sites can serve as both early-warning indicators and amplifiers of infectious disease risk. In this context, leveraging recent advances in technology, such as remote sensing, next-generation sequencing techniques, and ecological modelling, offers a timely opportunity to shift from reactive crisis response to proactive disease prevention.

Integrating carcass-based surveillance into existing frameworks will require sustained interdisciplinary collaboration among ecologists, epidemiologists, biologists, wildlife managers, microbiologists, and data scientists. Such partnerships are essential to refine field methodologies (carcass swabbing, environmental sampling, and camera trap deployment) and to ensure that the vast datasets they generate are processed through robust and scalable analytic pipelines. We acknowledge the existing logistical challenges in data sharing and integration in transdisciplinary contexts (97), and draw attention to the ‘One Health Joint Plan of Action’, which outlines guidelines for effective data integration in One Health surveillance systems, such as the one proposed here (6). Strengthening collaborations between diverse disciplines is fundamental to embedding carcass surveillance within operational One Health frameworks and ensuring that findings are translated into coordinated, cross-sectoral action. Whilst full discussion of these challenges is beyond the scope of this manuscript, as surveillance methods develop at this One Health interface, future research should prioritise mechanisms for co-design, standardisation, and shared governance to operationalise carcass-based surveillance within national and regional health security systems.

Crucially, inter-disciplinary surveillance efforts must also account for the ecological complexity of the carcass–scavenger interface. Scavengers play a dual role in disease dynamics, functioning as both microbe removers and amplifiers that can facilitate ongoing transmission. This duality emerges from an interplay of processes including direct carcass consumption, environmental contamination, vector activity, host aggregation, and trophic interactions, that vary across space, time, and species. Designing effective surveillance and management strategies thus demands not only technical innovation but also ecological insight, so that disease risk can be mitigated without compromising the vital role scavengers play in ecosystem functioning.

From an ecological perspective, the necrobiome (the microbial communities involved in carcass decomposition) remains poorly understood. Building surveillance systems around carcasses must therefore go together with foundational research that contextualises necrobiome composition and function across different habitats, climates, and decay stages. To achieve this, it is essential to integrate diverse methodologies that can capture the full complexity of scavenger–carcass dynamics. The combination of ecological fieldwork with remote sensing tools (such as camera traps and GPS telemetry), molecular techniques, and network analysis enables insight into how pathogens flow through wildlife communities to produce data that can inform One Health strategies, strengthen surveillance programs, and guide ecosystem management in both routine and crisis settings. However, selecting the most appropriate tools and analyses depends on clearly defined goals. Surveillance aimed at early outbreak detection may require high-frequency sampling and real-time data processing, whereas broader ecosystem health assessments might prioritise periodic microbial profiling or spatially explicit modelling.

Looking ahead, shifting species distributions, altered mortality patterns, and changing scavenger behaviour will continue to reshape the disease ecology. Carcasses will play an increasingly prominent role in pathogen dissemination across wildlife, domestic animals, and even humans. Recognising this, we must identify microbial assemblages and transmission routes associated with carcass sites, both to improve our understanding of natural decomposition processes and to develop carcass-based surveillance for disease prevention globally. By investing in this emerging field, we can enhance early-warning capabilities, support biodiversity, and build more resilience at the human–animal–ecosystem interface.

## Data Availability

The original contributions presented in the study are included in the article/supplementary material, further inquiries can be directed to the corresponding author/s.
